# The mother of all actins?

**DOI:** 10.7554/eLife.23354

**Published:** 2016-12-20

**Authors:** Felipe Merino, Stefan Raunser

**Affiliations:** 1 Department of Structural Biochemistry, Max Planck Institute of Molecular Physiology, Dortmund, Germany; 1 Department of Structural Biochemistry, Max Planck Institute of Molecular Physiology, Dortmund, Germanystefan.raunser@mpi-dortmund.mpg.de

**Keywords:** *Pyrobaculum calidifontis*, F-actin, bacterial cytoskeleton, Crenarchaea, MreB, arcade cluster, Other

## Abstract

New insights into the structure of filaments made of crenactin, a homolog of actin found in archaea, shed light on how the cytoskeleton might have evolved.

**Related research article** Izoré T, Kureisaite-Ciziene D, McLaughlin SH, Löwe J. 2016. Crenactin forms actin-like double helical filaments regulated by arcadin-2. *eLife* **5**:e21600. doi: 10.7554/eLife.21600

There was a time when most scientists believed that eukaryotes had cytoskeletons and that bacteria and archaea did not. This view changed in the early 1990s when a bacterial protein – that was later identified as a tubulin homolog – was shown to form a ring-like structure that is essential for bacterial cells to divide (Bi and Lutkenhaus, 1991; Mukherjee and Lutkenhaus, 1994). It has since been discovered that bacteria and archaea carry homologs for all the components of the eukaryotic cytoskeleton – actin, tubulin and intermediate filaments (Shih and Rothfield, 2006). What is more, we now know that many of these homologs form complex filamentous structures and fulfill similar roles to the cytoskeleton of eukaryotes. This indicates that the cytoskeleton most likely evolved further back in the history of life on Earth than originally thought.

The first eukaryotic cells are thought to have originated after an archaeal cell engulfed an ancient bacterium and the two established a symbiotic relationship (Rivera et al., 1998; Yutin et al., 2008). An archaeon called *Pyrobaculum calidifontis* is believed to be one of the closest living relatives of the ancestral archaea involved in this event (Guy and Ettema, 2011), and has been studied by many researchers. A key component in the cytoskeleton of *P. calidifontis* – a protein called crenactin – is a homolog of actin. While crenactin is only ~20% identical to eukaryotic actin, of all the proteins found outside of the eukaryotes, it is the one that is most closely related to actin (Ettema et al., 2011).

Two years ago, Jan Löwe, Thierry Izoré and colleagues at the MRC Laboratory of Molecular Biology reported that the crystal structure of crenactin was similar to that of eukaryotic actin in many respects (Figure 1). However, while actin forms double-stranded filaments, crenactin appeared to form only single-stranded helices (Izoré et al., 2014). Using electron cryo-microscopy, another group later reported that crenactin also forms single-stranded helices in solution (Braun et al., 2015).

**Figure 1.**
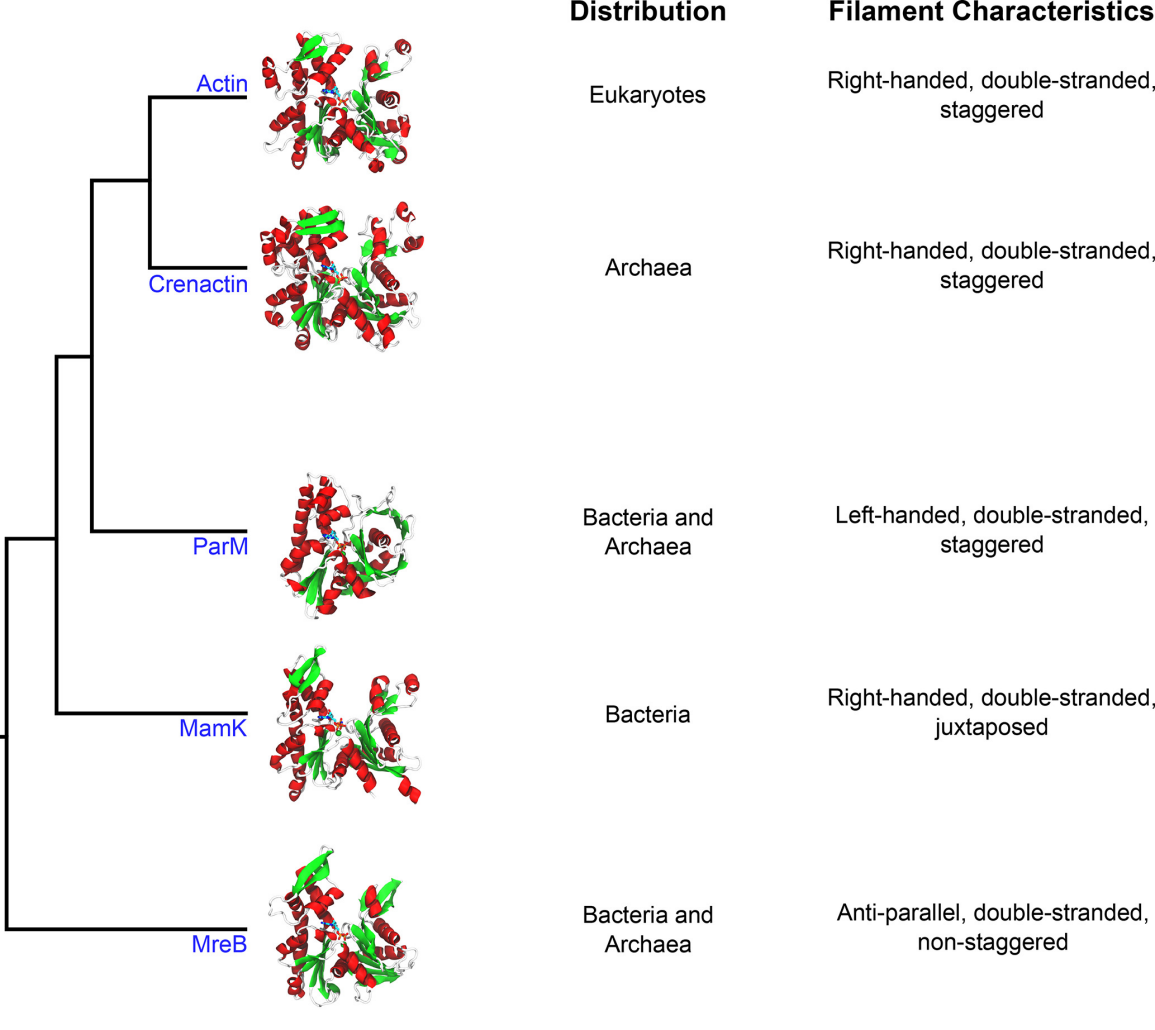
Crenactin is a member of the actin superfamily. Representative structures of individual proteins (in the non-polymerized conformation) belonging to each family illustrate the overall similarity between the proteins. The domains of life where homologs for these proteins have been identified are listed in the first column (distribution), and the properties of filaments made of each protein are listed in the second column (filament characteristics). The tree diagram was modified from Ettema et al. (2011). The structures use the following PDB entries: 1J6Z (actin), 5LY3 (crenactin), 4A62 (ParM), 5LJK (MamK) and 4CZL (MreB).

Now, in eLife, Löwe and colleagues – including Izoré as first author – have used electron cryo-microscopy to further investigate the structure of crenactin filaments (Izoré et al., 2016). However, contrary to the previous reports, Izoré et al. now show that crenactin filaments are more similar to actin filaments than previously thought. Indeed, both form parallel, double-stranded filaments with a similar helical arrangement, and the individual subunits in a crenactin filament interact like those in an actin filament.

Izoré et al. note that the earlier studies used salt concentrations much higher than the concentrations that *P. calidifontis* needs to grow. Although the salt concentration inside this bacterium is not known so far, they suggest that this could have interfered with how the filaments formed and could thus explain the discrepancy between the previous and new results. The double-stranded filaments also seem to be more stable than the single-stranded crenactin filaments. This meant that Izoré et al. could, for the first time, study crenactin filaments in enough detail to be able to distinguish their finer features.

The strong resemblance between actin and crenactin suggests that actin has looked much like it does today since early on in the evolution of eukaryotes. Interestingly, actin is required for phagocytosis, the process by which a eukaryotic cell engulfs a bacterium. As mentioned above, it is thought a similar mechanism gave rise to the first eukaryotes (Yutin et al., 2009), and the presence of a working actin-like cytoskeleton in the ancestral archaea would certainly support this idea.

The gene encoding crenactin is part of a group of five genes called the arcade cluster that are found within the genomes of the Crenarchaeota (Ettema et al., 2011). This is the group of archaea to which *P. caldifontis* belongs and which gives crenactin its name. In *P. caldifontis,* three proteins encoded by other genes in the arcade cluster – called arcadin-1, -3, and -4 – localize with crenactin filaments inside cells, hinting that they might interact. In contrast, arcadin-2 does not co-localize with filaments (Ettema et al., 2011).

Izoré et al. also report that the C-terminal region of arcadin-2 strongly interacts with individual crenactin proteins, effectively sequestering them and preventing them from polymerizing to form filaments. In turn, this explains why arcadin-2 has a different distribution pattern to the other arcadins. Izoré et al. then went on to show, using X-ray crystallography, that a fragment of arcadin-2 encompassing its C-terminal portion folds into a helix and binds to crenactin. The binding site corresponds to a site on actin that is used by eukaryotic proteins involved in disassembling filaments.

Further experiments showed that arcadin-1 interacted very poorly with the individual crenactin proteins. However, considering its distribution in the cell, it is still possible that arcadin-1 prefers to interact with filaments rather than individual proteins.

Izoré et al. report that the structure of arcadin-1 does not look like any other known protein structure, meaning that it represents a new protein fold. They also note that the primary sequence of arcadin-2 is unlike that of any actin regulator known to date. This suggests that while the actin cytoskeleton might predate the first eukaryotes, its regulation has been invented independently during evolution. The fact that several, evolutionarily-unrelated proteins use similar mechanisms to fine-tune the polymerization state of actin provides further support for this idea.

All in all, the discoveries of Löwe, Izoré and colleagues highlight the remarkable level of complexity in the cytoskeletons of non-eukaryotes. Moreover, the continuous development of microbiology guarantees that this picture is going to become ever more complex. Hopefully, these advances will bring us even closer to understanding the evolution of the cytoskeleton.
